# Monitoring the Occurrence of *Aspergillus* in the Air of Intensive Care Units

**DOI:** 10.3390/microorganisms13051099

**Published:** 2025-05-09

**Authors:** Anna Inglot, Agnieszka Gniadek, Zuzanna Tokarz, Wirginia Krzyściak, Monika Papież, Paweł Krzyściak

**Affiliations:** 1 Postgraduate Student at the Faculty of Health Sciences, Jagiellonian University Medical College, Kopernika Street 45, 31-501 Krakow, Poland; 2 Department of Nursing Management and Epidemiological Nursing, Institute of Nursing and Midwifery, Faculty of Health Sciences, Jagiellonian University Medical College, Kopernika Street 45, 31-501 Krakow, Poland; agnieszka.gniadek@uj.edu.pl; 3 Department of Infection Control and Mycology, Chair of Microbiology, Faculty of Medicine, Jagiellonian University Medical College, Czysta Street 18, 31-008 Krakow, Poland; zuzanna.bebenek@uj.edu.pl; 4 Department of Medical Diagnostics, Faculty of Pharmacy, Jagiellonian University Medical College, Medyczna Street 9, 30-688 Krakow, Poland; wirginia.krzysciak@uj.edu.pl; 5 Department of Cytobiology, Faculty of Pharmacy, Jagiellonian University Medical College, Medyczna Street 9, 30-688 Krakow, Poland; monika.papiez@uj.edu.pl

**Keywords:** *Aspergillus*, molds, CAPA, indoor air quality, ICU, hospital

## Abstract

Poor air quality within hospitals can contribute to a range of health issues, collectively known as sick hospital syndrome, encompassing respiratory, skin, and nonspecific symptoms. *Aspergillus* poses a significant risk of severe respiratory infections, particularly within intensive care unit (ICU) patients often with compromised immune systems. This study was conducted in the intensive care units of three selected hospitals in Cracow, Poland. Air samples were obtained using the single-stage impactor MAS-100 NT Ex (Merck). The air samples were taken from each ward, seasonally, four times a year. Sampling of intensive care units’ air for *Aspergillus* revealed the significant diversity in fungal concentration and unveiled seasonal fluctuations in culturable fungi levels. The highest concentration of *Aspergillus* species complexes was detected during autumn, while the lowest was in spring. The disturbing occurrence of *Aspergillus* in hospitals’ air emphasizes the importance of monitoring fungal air bioburden and assessing air control efficiency and highlights the urgent need to develop and validate microbiological standards for fungal air contamination in hospitals.

## 1. Introduction

A fungal aerosol is a complex matrix consisting of various fungal elements, including mycelial fragments, hyphae, or spores [1]. The bioaerosol may include dormant, culturable, viable, or dead fungal cells. The types, sizes, and concentrations of fungal particles in the air depend on environmental factors such as climate, weather, temperature, humidity, air currents, time of the day and year, age of the building, indoor traffic, type, and maintenance of ventilation systems [1,2]. Numerous medical procedures are prone to generating aerosols of small particle sizes, which pose an inhalation risk for patients and healthcare workers who may be exposed [3]. *Aspergillus* infections are the second-most common nosocomial fungal infections and cause 1.3% of all iatrogenic mycosis [4,5]. Immunocompromised patients, particularly those in intensive care units, are highly susceptible to invasive aspergillosis. Major host-related risk factors include prolonged neutropenia (absolute neutrophil count <500 cells/μL for ≥10 days), oral or parenteral corticosteroid therapy equivalent to ≥0.3 mg/kg/day of prednisone for ≥3 weeks, and immunosuppressive regimens used in solid organ or hematopoietic stem cell transplantation. These conditions impair pulmonary innate immunity and facilitate fungal spore germination, hyphal growth, and tissue invasion.

A positive correlation exists between the level of fungal spores in the air and the risk of infection or allergic reactions [2,6]. Sources of *Aspergillus* fungi infection may include other infected individuals or the hospital environment: air, water supply systems, ventilation systems, hospital food, or medical equipment [4,7]. Common entry points of *Aspergillus* include the patient’s respiratory system and areas of impaired skin integrity, e.g., burns, postoperative wounds, or corneal damage. Infection in the respiratory system develops by the aspiration of fungal spores suspended in the air. The fungi growing in the lungs (incubation period is 2 days to 3 months) cause bleeding, which can contribute to further transmission of the infection through the bloodstream to the brain, liver, spleen, kidneys, pericardium, or skin, resulting in life-threatening invasive aspergillosis (IA) [8]. Pulmonary diseases, such as cystic fibrosis (CF), chronic obstructive pulmonary disease (COPD), or viruses and bacterial infections, impair the respiratory system’s immune defense and promote mycosis [9]. The risk of aspergillosis among ICU patients is between 0.33 and 5.8% and 5–10 times higher than among other patients [10,11]. In a study conducted in the ICU of one Belgian hospital, *Aspergillus* infection was detected in 7% of the patients, and the mortality rate reached 91%. Interestingly, 70% of these patients had no risk factors for invasive fungal disease [12]. According to the National Guidelines for the Prevention of Nosocomial Aspergillosis (2018) [13], mechanically ventilated patients are categorized as patients with an increased risk for invasive aspergillosis, whereas neonates in the ICU are in a high-risk group. Immunological immaturity makes neonates extraordinarily susceptible to nosocomial infections [14]. Research shows that the nosocomial fungal infection rate among neonates reaches 12–16% worldwide [15]. Critically ill patients with compromised pulmonary defenses, pulmonary inflammation, and ventilator dependence, particularly those with widespread respiratory virus infections, especially influenza-A virus and SARS-CoV-2, are reported to be at risk for invasive pulmonary aspergillosis (IPA) [16,17,18]. Both coinfections—COVID-19-associated pulmonary aspergillosis (CAPA) and influenza-associated pulmonary aspergillosis (IAPA)—result in poor prognosis and high mortality. The prevalence of CAPA has been estimated between 5% and 15% and IAPA between 10% and 32%, and the mortality reaches 50% for CAPA cases and 60% for IAPA [18]. *Aspergillus* spp. has been classified as a critical priority fungal pathogen by the World Health Organization, primarily due to its clinical impact and the emergence of antifungal resistance.

The increase in fungal infections with a high morbidity and mortality rate among hospital patients emphasizes the importance of air quality monitoring [10]. Routinely performed airborne fungi concentration monitoring allows us to determine and minimize the risk of mycosis for hospitalized patients. This study aims to determine the level of air microbiological contamination with mold, with particular emphasis on *Aspergillus* in ICUs, taking into consideration the impact of seasonal fluctuation, temperature, and humidity.

## 2. Materials and Methods

### 2.1. Sampling Areas

This study was conducted in the intensive care units of selected hospitals in Cracow. The hospitals included in this research were chosen to ensure diverse technical conditions, including building age, equipment, presence and quality of the air conditioning system, and geographic location, while maintaining the same specificity of the wards (intensive care units only) and comparable purposes of the rooms from which the samples were obtained. The hospital building in which the first intensive care unit (ICU 1) is located was commissioned in 2019. Hospital number 2 was built in 1954, but the intensive care unit (ICU 2) was renovated in 2015. Hospital number 3 is located in a building from 1925, and the researched unit (ICU 3) was renovated in 2017. Both ICU 1 and ICU 2 are general intensive care units, and ICU 3 is a neonatal intensive care unit. Patient rooms in ICU 1 and ICU 3 have mechanical ventilation systems equipped with HEPA filters. In the corridors of ICU1 and ICU 3, gravitational ventilation is used. In ICU 2, a ventilation system is present; however, we were denied access to detailed information about its type and the filters used. The criterion for including rooms in this study was patients’ unavoidable contact with the surrounding air: in front of the entrances to wards and rooms, patient transfer areas, corridors, patient rooms, isolation rooms, treatment rooms, the nurse module, and outside of the building. Due to COVID-19 pandemic restrictions, we were only allowed to sample the air of isolation rooms in ICU 1.

### 2.2. Air Sampling Procedure

The air samples were obtained using the single-stage impactor MAS-100 NT Ex (Merck KGaA, Darmstadt, Germany) [19]. The MAS-100 device meets the requirements of The European Union. The sampling process adhered to ISO 16000-18:2011 guidelines [20]. The resulting air stream, carrying particles, is directed to the surface of the Saburaudculture media [4% dextrose agar] (SGA, prepared in our laboratory from peptone special (Oxoid, Basingstoke, UK), glucose (Chempur, Piekary Slaskie, Poland), agar (Biomaxima, Lublin, Poland), and chloramphenicol (Pol-Aura, Dywity, Poland)) and Rose Bengal media (RBA, Oxoid, Basingstoke, UK) in a standard Petri dish. Both media were used in parallel to allow enumeration of a broad spectrum of airborne fungi. Rose Bengal Agar, due to the presence of Rose Bengal dye, limits colony size and spreading, which facilitates counting, especially of slower-growing or xerophilic species.

The total volume of air per sample was 200 L/2 min. The air samples were taken from each ward, seasonally, four times a year: in May 2021, August 2021, November 2021, and March 2022. Sampling was conducted 1.5 m above the ground under operational conditions of peak staff activity, starting at 7 a.m. each day. Due to the lack of guidelines as to the volume of air in aeromycological tests, for this project, we adopted the principle of taking 30% of the total air volume from each chosen room. The cubic capacity of each room was measured with a laser distance meter Uni-T LM60 (Uni-Trend Technology Co., Ltd., Dongguan, China). The temperature (in °C) and humidity (in %) were taken with a thermo-hygrometer PWT-103 (Elmetron, Zabrze, Poland). Measurements were made three times in each room, in places farthest from the sources of air supply and free from intensive air exchange (avoiding open doors, windows, and drafts), immediately before each sampling. Each result was recorded, and the arithmetic mean was calculated.

### 2.3. Fungi Enumeration and Identification

The collection of materials followed strict aseptic procedures, with samples transported to the laboratory within one hour. Petri dishes were incubated at 25 °C ± 2 °C for seven days, after which fungal colonies were counted and identified. Enumeration was performed by counting colonies on the medium and expressing the results in Colony-Forming Units (CFUs) per 1 m^3^ of air, adjusted using the Feller formula for the MAS-100 NT Ex (Merck KGaA, Darmstadt, Germany) instrument (SH 400 holes of 0.7 mm diameter each). *Aspergillus* isolates were further subcultured on Czapek Yeast Agar (CYA prepared in our laboratory from Czapek Dox Modified Agar (Oxoid^TM^, Basingstoke, UK), Yeast Extract (Sigma-Aldrich, Steinheim, Germany), and chloramphenicol (Pol-Aura, Olsztyn, Poland)) to enhance sporulation for detailed morphological assessment, facilitating identification to the subgenus and section levels by a qualified mycologist (PK). Additionally, 10% of randomly selected strains underwent confirmation using MALDI-TOF analysis (Figure S1).

### 2.4. Data Analysis

The fungal colonies’ growth on the medium was counted and expressed in Colony-Forming Units (CFUs). The number of Colony-Forming Units was corrected for the MAS-100 instrument according to the Feller formula for SH 400 holes of 0.7 mm diameter each [19]. The total number of fungi in 1 m^3^ (X) was calculated according to the formula X = (a × 1000)/V, where a is the sum of *Aspergillus* colonies that grew on the Petri dishes, and *V* is the volume of the sampled air in liters. To assess inter-seasonal fluctuation (ISF) in Aspergillus spp. counts, we calculated the mean and standard deviation of absolute differences (|Δ|) between consecutive seasons (spring→summer, summer→autumn, autumn→winter, winter→spring) for each ICU.

### 2.5. Statistical Analysis

We used the non-parametrical Kruskal–Wallis test to compare mold concentration levels between researched hospitals, locations, temperature, and humidity. For further statistical analysis, statistically significant results were tested with Dunn’s Kruskal–Wallis multiple comparison test. If no statistically significant differences were found, we used the mean value of CFUs grown on SGA and RBA media, obtained from the same location.

## 3. Results

*Air Contamination*

In the four seasons, we obtained 448 air samples from three intensive care units from different hospitals. From ward number 1 (ICU 1), we obtained 186 samples, ward number 2 (ICU 2) 140 samples, and ward number 3 (ICU 3) 122 samples. The number of detected CFUs was estimated from 0 (multiple locations) to 430 CFU/m^3^ (patient room ICU 3) (Table 1).

From all 448 collected air samples, 135 plates (30.13%) showed no growth of fungal colonies: 90 from the patient rooms, 18 from the corridors, 1 from the front of the entrance location, and 5 from the isolation rooms. We observed mold growth in various concentrations on the 313 remaining plates (69.87%). The total number of 796 *Aspergillus* colonies were observed on 201 plates (43.31%). We identified four subgenera and nine sections of Aspergillus spp. The subgenus *Nidulantes* included the sections *Versicolores*, *Nidulantes,* and *Usti* (358, 44.9%). The subgenus *Fumigati comprised* the sections *Fumigati* and *Clavati*. The subgenus *Circumdati* included the sections *Circumdati* (16.21%), *Nigri* (5.65%), and *Flavi* (1.63%). Finally, the subgenus *Aspergillus* was represented by the section *Aspergillus* (3.64%). The complete data are presented in Table 2.

Of the samples, 43.60% were from patient rooms, 41.46% from corridors, 54.16% from the front of the entrance, and none from the isolation rooms. Mean values and ranges of *Aspergillus* CFU concentrations are shown in Table 3.

The highest number of *Aspergillus* species complex colonies was observed in ICU 3, with mean CFU numbers of 25.00 CFU/m^3^ in front of the entrance, 7.00 CFU/m^3^ in patient rooms, and 27.50 CFU/m^3^ in corridors. The most abundant growth of *Aspergillus* colonies was observed in autumn with a mean of 3.45 CFU/m^3^. The lowest number of *Aspergillus* colonies was observed in spring, with a mean value of 0.67 CFU/m^3^. A statistically significant difference between *Aspergillus* occurrence and seasons was found in ICU 2 (Kruskal–Wallis chi-squared = 22.899, df = 3, *p*-value = 4.239 × 10^−5^) and ICU 3 (Kruskal–Wallis chi-squared = 28.994, df = 3, *p*-value = 2.246 × 10^−6^). It is worth mentioning that no *Aspergillus* colonies grew on the plates from ICU 1 in autumn and winter. More specific data are described in Table 4.

ISF in Aspergillus spp. counts was most pronounced in ICU 3, indicating strong and irregular seasonal variation. ICU 2 showed moderate fluctuation, while ICU 1 remained relatively stable throughout the year. The largest seasonal shifts were observed between summer and autumn, and between autumn and winter, particularly in ICU 3 (Table 4).

There were no statistically significant correlations between the concentration of *Aspergillus* molds and temperature. However, both humidity and the specific hospital were significant factors influencing *Aspergillus* levels (multivariate analysis). Notably, statistical significance for these effects was observed only in summer.

## 4. Discussion

Research on the occurrence of *Aspergillus* in the air of ICUs has been conducted in various regions around the world to determine the prevalence of *Aspergillus* in ICU environments, evaluate the potential risk of infection, and identify potential sources of contamination. Several studies have been conducted to determine the prevalence and significance of *Aspergillus* species complexes in the air of ICUs. *Aspergillus* is a type of fungi commonly found in the environment, and it can pose a significant risk to patients with weakened immune systems, such as those in ICUs. Research shows that airborne fungal spores in hospitals are not only a theoretical risk—they have been directly linked to patient infections by isolating and genetically matching strains from both environmental and clinical samples. Warris et al. found that air and patient isolates of *A. fumigatus* were genetically indistinguishable [21]. In a hematology ward, using molecular typing, they confirmed that airborne strains were genetically identical to clinical isolates from patients with IA, directly linking air contamination to nosocomial infection [22].

In the present study, mold numbers ranged between 0 CFU/m^3^ and 430 CFU/m^3^, and *Aspergillus* propagule numbers varied from 0 CFU/m^3^ to 27.5 CFU/m^3^, according to the hospital. Another study conducted in the intensive care unit, neurosurgery unit, and bone marrow transplant unit in China showed that the mean concentration of fungi in the air of the abovementioned wards was, depending on the ward, between 71.02 and 91.94 CFU/m^3^ [23]. A Belgian study conducted in the ICU found that the mean number of fungal colonies in the air was between 16 and 100 CFU/m^3^. In a study conducted by Martinez-Herrera et al. [24], the highest fungal air contamination in ICU air was 43.51 CFU. However, a study from Thailand showed that the mean fungal colonies in the air of ICUs varied from 80 to 215.7 CFU/m^3^, with *Aspergillus* prevalence in fungal bioaerosol (48.6% of all found fungi) [25]. In our study, significantly higher levels of Aspergillus CFUs compared to other similar studies were observed in ICU 2, which is located in a building near green areas. The location of the building may have contributed to the high level of *Aspergillus* CFUs due to the wind dispersing mold spores, contaminating the hospital air.

The diversity of *Aspergillus* species complex concentration may suggest a significant problem with mold spreading in hospital environments and difficulties in maintaining air cleanliness in hospitals worldwide. It is particularly important given the risk factors for IPA, such as rampant viral respiratory infections including influenza, parainfluenza, SARS-CoV 2, and respiratory syncytial virus, as well as COPD and asthma, common among ICUs’ patients [26]. In our study, the most common *Aspergillus* species belonged to the section *Versicolores* (43%), followed by species from the sections *Fumigati* and *Circumdati*. In a Brazilian study, *Aspergillus niger* was the most dominant species, identified in 60% of indoor air isolates from public and private hospitals [27]. Similarly, *Aspergillus niger* was the most frequently reported species in a Turkish investigation, representing 29.1% of all detected isolates [28]. In contrast, in a study conducted by Martinez Herrera et al., the most prevalent Aspergillus species were *A. fumigatus* and *A. flavus* [24].

In the recent literature, no unequivocal recommendation exists on the acceptable level of fungi concentrations in the air of intensive care units. According to Krzysztofik [29], the permissible level of fungal air contamination on Sabouraud medium is 0 CFU/m^3^ for operating rooms, 5 × 10^1^ CFU/m^3^ for treatment rooms, and 2 × 10^2^ CFU/m^3^ for patients rooms. However, there are no specific guidelines for ICU rooms.

The EU-WHO (European Union–World Health Organization partnership) standards for hospital room air contamination classify microbiological air quality into three categories based on patient risk [30]. According to these standards, pediatric, general, and surgical intensive care units fall within class II, with a microbiological air contamination limit of < 50 CFU/m^3^. Nonetheless, these standards apply to general microbial air contamination without specifying the acceptable concentration of fungi.

The Spanish Society of Infectious Diseases and Clinical Microbiology (SEIMC—Sociedad Española de Enfermedades Infecciosas y Microbiología Clínica) (2011) [31] published guidelines on the prevention of invasive mold diseases caused by filamentous fungi, determining counts above 25 CFU/m^3^ as dangerous in hospital settings. However, for protected rooms, such as isolation rooms for patients with immunodeficiency, and operating areas, the limit of 0.5 CFU/m^3^ of air was established.

In the present study, 10.04% of samples (45 plates) exceeded the limit of 200 CFU/m^3^ by Krzysztofik [29], and 36.16% of samples (162 plates) showed growth more abundant than 50 CFU/m^3^, exceeding the conformity criteria set by EU-WHO [30].

In Poland, there are no available guidelines for acceptable *Aspergillus* counts. However, it is crucial to assess whether the fungal air concentration in hospitals is safe for patients or not. The Irish National Guidelines for the Prevention of Nosocomial Aspergillosis (2018) [13] has established *Aspergillus* concentration threshold levels, acceptable in hospital settings. For HEPA (High-Efficiency Particulate Air)-filtered air, the maximum allowable *Aspergillus* air contamination was determined to be <1 CFU/m^3^, and <5.0 CFU/m^3^ in units with no air filtration. However, Risk of fungal infections and construction work in hospitals. Identification of risks and implementation of management precautions by the French Society for Medical Mycology (SFMM—Société Française de Mycologie Médicale) and the French Society for Hospital Hygiene (SF2H—Société Française d’Hygiène Hospitalière) (2011) [32] recommends that for protected environments (including areas and rooms with HEPA-filtered air), there should be no *Aspergillus* CFUs, and for other hospital areas, acceptable *Aspergillus* concentrations should not be greater than 2 CFU/m^3^. Nonetheless, a study conducted in Thailand revealed that *Aspergillus* species complex concentrations of up to 10 CFU/m^3^ in the air are associated with a low risk of invasive aspergillosis [25]. Another study conducted by Pelaez et al. [33] reported that a concentration of *Aspergillus fumigatus* spores above 17.4 CFU/m^3^ increases the risk of invasive aspergillosis among immunocompromised patients in the intensive care unit. The lack of explicit recommendations makes it difficult to provide a safe condition for patients and prevent *Aspergillus* infections.

One study suggests that climatic conditions can influence the fungal air population [34]. Hai et al. [35] confirmed the strong correlation between fungal levels and environmental conditions such as temperature and humidity. Most *Aspergillus species complexes* develop at a minimum temperature of 10–25 °C to a maximum of 35–55 °C and at air humidity levels above 45% [36]. According to Polish standards, in ICUs, the air temperature should be in the range of 22–26 °C, and the recommended level of air humidity is 40–60% [37]. The present measurements (mean humidity 40.58%, mean temperature 23.51 °C) comply with the required conditions. These results provide excellent conditions for *Aspergillus* expansion, which was demonstrated by the observed correlation between *Aspergillus* propagule concentration and humidity and temperature. However, in a research project performed by Hamzah et al. [38], despite measured temperatures ranging from 29.6 °C to 32.3 °C and humidity from 41% to 59%, creating perfect conditions for fungi growth, they found no statistical significance between either temperature or humidity and *Aspergillus* abundance. Puhlmann et al. [39] also did not establish a clear correlation between humidity or temperature and the seasonal air load of molds.

In their research, Krogulski and Podsiadły [37] observed a rapid increase in the number of fungi and their spores in the air, from April to the beginning of July. Similar short-term changes in the air concentrations of individual fungal species complexes have been repeatedly observed [38,40]. Van Rhijn et al. [41], in their study, showed seasonal fluctuations in fungi in the air, with the *Aspergillus* spore counts peaking during the summer and autumn (from the end of May to October), with a CFU mean of 19.93 (SD 23.64). Savković et al. [42] reported a similar seasonal appearance of *Aspergillus* species complexes. Our data demonstrate that *Aspergillus* was the most abundant in autumn (mean 3.45 CFU/m^3^) and the least abundant in spring (mean value of 0.67 CFU/m^3^). However, the mean mold CFU count was the highest in summer (mean 318.82 CFU/m^3^) and the lowest in winter (mean 17.23 CFU/m^3^).

However, a recent Polish study by Górzyńska et al. [43] reported a peak in *Aspergillus* spore concentrations during spring (up to 300 CFU/m^3^), which contrasts with our findings [43]. The discrepancy between the results of Górzyńska et al. [43] and ours may be due to two reasons, including the fact that air samples were not collected cyclically in each season (only in spring and winter, so it is difficult to estimate the increase above the norm when the studies included only two measurements). In addition, the researchers did not monitor the environment surrounding the ward during the measurement of the presence of fungi, which does not exclude the possibility of renovations being carried out in the hospital itself or its vicinity. Also, importantly, it was noted that the building in which the studies were conducted was 120 years old, which significantly increases the risk of contamination with spores of fungi of the *Aspergillus* genus. These results could also be completely accidental, as it has been proven that *A. fumigatus* spores can temporarily increase their concentration to a significantly high level and then drop to a normal level within just 30 min [44]. Due to their hydrophobic surface, *A. fumigatus* spores are very effectively dispersed by air mixing [45]. It is very likely that the peak concentrations of *Aspergillus* spores were often missed during monitoring studies, which explains why such an increase in spores could have occurred in the study by Górzyńska et al. [43]. Research conducted by Oberle et al. [46] showed a temporary deviation in the number of *Aspergillus* spores in air samples. The *Aspergillus* peak with a median of 148 spores/m^3^ occurred shortly following the removal of Christmas trees, decorations, and cleaning activities. The increase in spore counts was incidental. The air sampling conducted 48 h later indicated a *Aspergillus* spore concentration of three spores per cubic meter at each sampling location. This study confirms Górczyńska et al.’s [43] results that activities that stir the air may cause the outbreak of *Aspergillus* air contamination [46].

All the abovementioned results indicate that warmer months are associated with higher abundance of *Aspergillus* species complex colonies. What is interesting in our findings is the fact that the highest *Aspergillus* species complex concentration peak was observed in the season with the lowest mean temperature (22.86 °C), despite the increased temperature being correlated with more abundant spores in the air [41].

One limitation of this study is the lack of molecular identification of the isolated *Aspergillus* species. However, the morphological and culture-based identification we performed enabled identification at the species complex level, which, although not equivalent to molecular resolution, is sufficient for assessing exposure to clinically relevant fungal groups. This approach allowed us to distinguish between less pathogenic complexes, such as *Aspergillus* subgenus *Versicolores*, and more pathogenic ones, such as *A. fumigatus*. In the context of our study, focused on environmental burden and potential patient exposure, this level of identification provides meaningful insight. As a future step, we are currently conducting antifungal susceptibility testing of the isolates using the EUCAST screening method, which will help identify potential azole resistance and further refine the clinical relevance of our findings.

## 5. Conclusions

The findings of this study indicate that ICU environments may be subject to significant airborne contamination with *Aspergillus* spp., posing a potential risk to immunocompromised patients. Given the susceptibility of this patient population to invasive aspergillosis, these results highlight the importance of regular aeromycological surveillance in critical care settings. Hospitals should consider re-evaluating their environmental control strategies to reduce fungal exposure and improve patient safety.

## Figures and Tables

**Table 1:** Total airborne mold contamination expressed as CFU per plate in different ICU areas.

		In Front of the Entrance	Corridors	Patient Rooms	Isolation Rooms
ICU 1	mean (sd)	71.88 (132.18)	10.26 (17.48)	2.42 (4.88)	0.83 (2.04)
	median (Q1–Q3)	8.75 (3.75–76.88)	2.50 (0.00–8.75)	0.00 (0.00–2.50)	NA
	range	0.00-270.00	0.00–55.00	0.00–25.00	0.00 5.00
ICU 2	mean (sd)	81.25 (38.60)	83.96 (51.79)	58.80 (43.94)	NA
	median (Q1–Q3)	77.50 (53.75–105.00)	82.50 (38.75–110.62)	52.50 (20.00–89.38)	NA
	range	42.50-127.50	12.50-170.0	0.00–157.50	NA
ICU 3	mean (sd)	146.88 (96.53)	135.75 (115.48)	164.95 (120.93)	NA
	median (Q1–Q3)	108.75 (84.38-171.25)	123.75 (33.12–246.88)	165.00 (43.75–276.25)	NA
	range	82.50–287.50	12.50–297.50	17.50–430.00	NA

NA—no available data.

**Table 2:** Identified *Aspergillus taxons* with total CFU counts and their relative percentages.

Subgenus	Section	Number of CFU	Percentage
*Nidulantes*	*Versicolores*	345	43.34%
	*Nidulantes*	12	1.51%
	*Usti*	1	0.1%
Subtotal		358	44.86%
*Circumdati*	*Circumdati*	129	16.21%
	*Nigri*	45	5.65%
	*Flavi*	13	1.63%
Subtotal		187	23.5%
*Fumigati*	*Fumigati*	138	17.34%
	*Clavati*	14	1.76%
Subtotal		152	19.1%
*Aspergillus*	*Aspergillus*	29	3.64%
Not recognized		70	8.79%
	Total	796	100%

**Table 3:** Airborne contamination with Aspergillus molds expressed as CFU per plate in different ICU areas.

	In Front of the Entrances	Corridors	Patient Rooms	Isolation Rooms
mean (sd)	2.08 (2.85)	2.02 (5.03)	1.69 (3.56)	0.00
median (Q1–Q3)	1.25 (0.00–2.00)	0.50 (0.00–2.00)	0.50 (0.00–2.00)	0.00
range	0.00–8.50	0.00–27.50	0.00–25.00	0.00

**Table 4:** *Aspergillus* spp. counts (CFU/plate) by season and ICU, with inter-seasonal fluctuation (ISF).

		Spring	Summer	Autumn	Winter	ISF |Δ| (Mean ± SD)
ICU 1	mean (sd)	0.19 (0.51)	0.30 (0.78)	0.00	0.00	0.15 (0.11)
	median (Q1–Q3)	0.00	0.00 (0.00–0.25)	0.00	0.00	
	range	0.00–2.00	0.00-3.5	0.00	0.00	
ICU 2	mean (sd)	0.37 (0.37)	2.11 (1.97)	1.68 (1.61)	1.58 (1.02)	0.87 (0.64)
	median (Q1–Q3)	0.50 (0.00–0.50)	1.50 (1.00–2.50)	1.50 (0.50–2.00)	2.00 (0.62–2.38)	
	range	0.00–1.00	0.00–8.50	0.00–7.50	0.00–3.00	
ICU 3	mean (sd)	2.00 (3.54)	2.00 (1.51)	12.37 (7.95)	1.88 (1.20)	5.37 (5.31)
	median (Q1–Q3)	1.00 (0.62–1.38)	1.50 (1.00–3.00)	12.50 (5.75–17.00)	1.50 (1.00–3.00)	
	range	0.00–12.00	0.00–5.50	2.00–27.50	0.00–4.50	

## Data Availability

The original contributions presented in this study are included in the article/Supplementary Material. Further inquiries can be directed to the corresponding authors.

## Supplementary Materials

The following supporting information can be downloaded at: https://www.mdpi.com/article/10.3390/microorganisms13051099/s1. Figure S1: Total mould CFU per plate across ICUs by season and location. Boxplots present the distribution of CFU counts in individual rooms within each ICU, including median, interquartile range, and minimum and maximum values for each season. Figure S2: *Aspergillus* CFU per plate across ICUs by season and location. Boxplots present the distribution of CFU counts in individual rooms within each ICU, including median, interquartile range, and minimum and maximum values for each season.
