# Supplementary material for: Monitoring the Occurrence of Aspergillus in the Air of Intensive Care Units
Source: Microorganisms. 2025 May 9;13(5):1099. doi: 10.3390/microorganisms13051099 (PMC12114014; doi:10.3390/microorganisms13051099)
Supplement: Supplementary file 1 [file microorganisms-13-01099-s001.zip › microorganisms-3544212-supplementary.pdf]

Suplementarny figures:

Monitoring the Occurrence of *Aspergillus* in the Air of Intensive Care Units

Anna Inglot [1*] †, Agnieszka Gniadek [2], Zuzanna Tokarz [3] †, Wirginia Krzyściak [4], Monika Papież [5],Paweł Krzyściak [3**] †

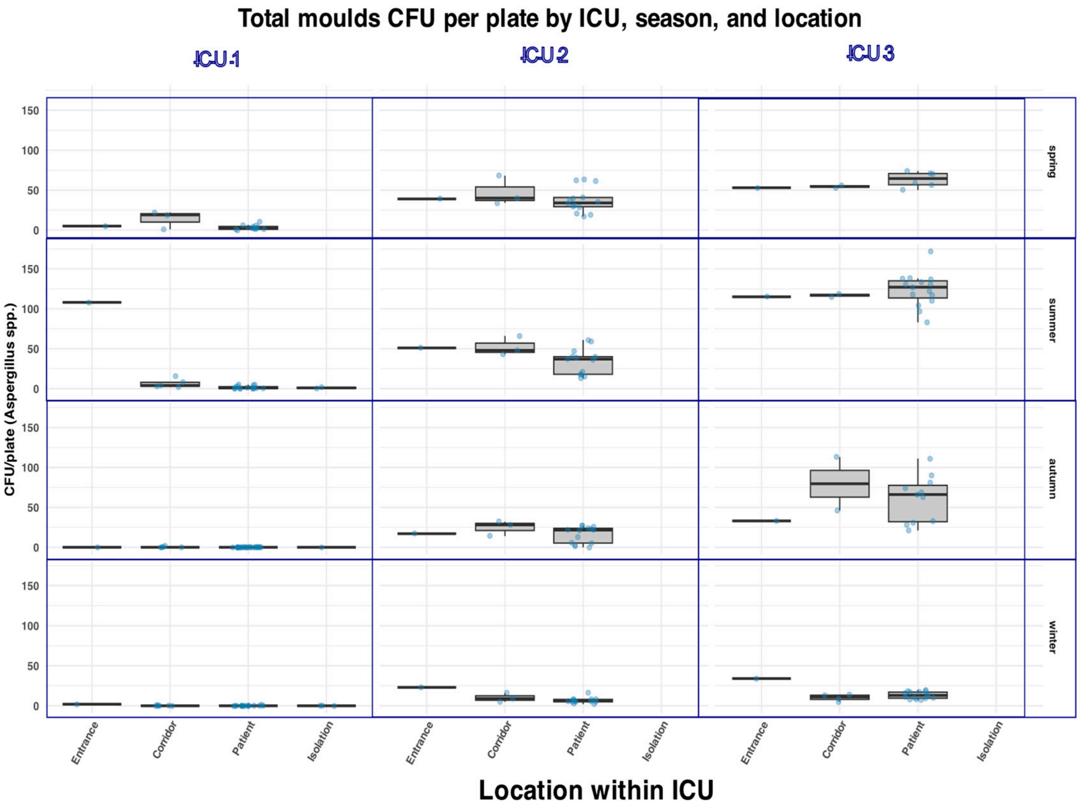

Figure S1. Total mould CFU per plate across ICUs by season and location. Boxplots present the distribution of CFU counts in individual rooms within each ICU, including

median, interquartile range, and minimum and maximum values for each season.

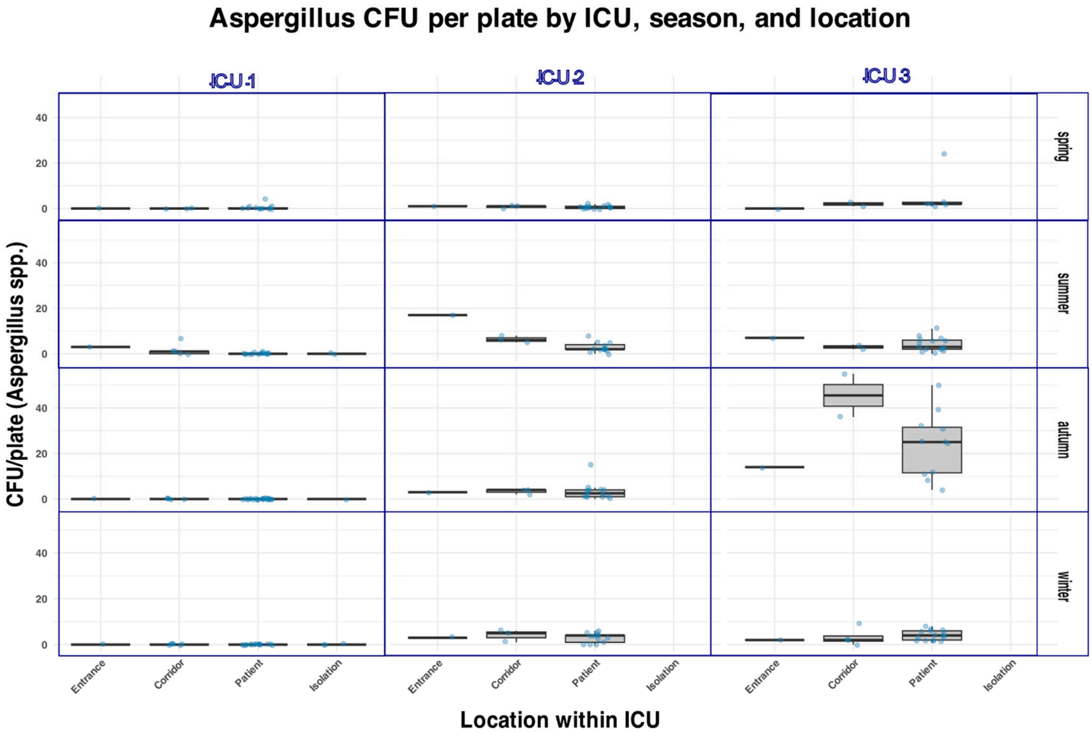

Figure S2. *Aspergillus* CFU per plate across ICUs by season and location. Boxplots present the distribution of CFU counts in individual rooms within each ICU, including median, interquartile range, and minimum and maximum values for each season.
